# *Salmonella enterica* subsp. *diarizonae* Harboring ST233, ST1263, and ST1845 in Children

**DOI:** 10.3389/fcimb.2021.727811

**Published:** 2021-08-19

**Authors:** Mingming Zhou, Qiucheng Shi, Xiucai Zhang, Lingling Mei, Yihua Ye, Chao Fang, Shiqiang Shang

**Affiliations:** ^1^Department of Clinical Laboratory, The Children’s Hospital, Zhejiang University School of Medicine, National Clinical Research Center for Child Health, Hangzhou, China; ^2^Microbiological Laboratory, Zhejiang Provincial Center for Disease Control and Prevention, Hangzhou, China

**Keywords:** *Salmonella enterica*, *diarizonae*, molecular epidemiology, resistance, pathogenicity, children, WGS

## Abstract

**Objective:**

This study aims to analyze the molecular epidemiology, resistance, and pathogenicity of *Salmonella enterica* subsp. *diarizonae* isolated from children.

**Methods:**

Whole genome sequencing was carried out, and molecular serotypes, sequence types, resistance genes, and virulence genes of *S. enterica* subsp. *diarizonae* isolates were analyzed. Antimicrobial susceptibility test was determined by commercialized microdilution method.

**Results:**

A total of three isolates of *S. enterica* subsp. *diarizonae* were isolated during 2015 to 2020. The molecular serotypes of the three strains were 61:c:z35, 61:l,v:1,5,7:[z57], and 65:k:z, respectively, and the sequence types were ST1845, ST233, and ST1263. All the three isolates were susceptible to ceftriaxone, ceftazidime, cefepime, amoxycillin/clavulanic acid, piperacillin/tazobactam, ertapenem, imipenem, levofloxacin, and trimethoprim/sulfamethoxazole. No other resistant gene was detected except *aac(6’)-Iaa*. There were no resistant plasmids detected in all the three isolates. A total of 76 genes were present in all isolates, containing 49 genes of Type III Secretion System (T3SS) mediated by SPI-1and SPI-2, 13 genes of adherence (type 1 fimbriae, Agf, and MisL-related genes), 11 genes of iron uptake (Yersiniabactin), two genes of magnesium uptake, and one gene of typhoid toxin(*cdtB*).

**Conclusion:**

The serotypes and sequence types of *S. enterica* subsp. *diarizonae* isolates were rarely reported in children; all the *S. enterica* subsp. *diarizonae* isolates were susceptible to detected antibiotics; T3SS, adherence, iron uptake, magnesium uptake, and typhoid toxin were responsible for pathogenicity of the *S. enterica* subsp. *diarizonae* isolates in children.

## Introduction

*Salmonella* is the predominant bacteria causing diarrhea in children, especially in infancy and early childhood, which seriously threatens the lives and health of children, and previous study has stated that there were over 93 million cases of gastroenteritis and 155,000 deaths caused by nontyphoidal *Salmonella* per year (Majowicz et al., 2010). The genus *Salmonella* includes only two species, *Salmonella enterica* and *Salmonella bongori*. *S. enterica* is further subdivided into six subspecies: *enterica*, *salamae*, *arizonae*, *diarizonae*, *houtenae*, and *indica* (Guibourdenche et al., 2010). Virtually all of *Salmonella* infections in humans were caused by the strain of *S. enterica* subsp. *enterica* (McClelland et al., 2001). *S. enterica* subsp. *diarizonae* is known to cause infections in ectothermic animals (Schroter et al., 2004). However, *S. enterica* subsp. *diarizonae* related cases in human have been gradually reported (Chong et al., 1991; Gerlach et al., 2017; Giner-Lamia et al., 2019; Uelze et al., 2020; Pan et al., 2021). Additionally, limited study has been reported in the area of molecular epidemiology, resistance, and pathogenicity of *S. enterica* subsp. *diarizonae* in children (Gerlach et al., 2017; Giner-Lamia et al., 2019). Whole genome sequencing (WGS) provided information regarding multilocus sequence typing (MLST), pathogenicity genes, and other contents (Kozyreva et al., 2016), which have rendered the systematic study of bacterial pathogens at the molecular level efficient and convenient. Accordingly, this study set out using WGS to determine the molecular epidemiology, resistance, and pathogenicity of *S. enterica* subsp. *diarizonae* in children from a tertiary university children’s hospital in China during 2015 to 2020.

## Methods

### Strain Collection and Identification

Three clinical *S. enterica* subsp. *diarizonae* isolates were collected at The Children’s Hospital, Zhejiang University School of Medicine from January 2015 to December 2020. First, fecal specimens were inoculated on SS medium (Comagal, Shanghai, China) and incubated for 18–24 h, while other specimens were inoculated on Columbia blood agar (Bioivd, Zhengzhou, China). The suspicious colonies of *Salmonella* were then identified using matrix-assisted laser desorption ionization time-of-flight mass spectrometry (MALDI-TOF MS, Bruker, Germany). *Salmonella* isolates were serotyped according to the modified Kauffmann*-*White scheme using commercial antisera (SSI, Copenhagen, Denmark) (Guibourdenche et al., 2010). *Salmonella typhimurium* ATCC14028 was used for quality control.

### Antimicrobial Susceptibility Test

Antimicrobial susceptibility test was determined by commercialized microdilution method (VITEK COMPACT, BioMérieux, Marcy-l’Étoile, France), and the results were interpreted according to the Clinical Laboratory Standards Institute (CLSI) guidelines M100-S30. Ceftriaxone, ceftazidime, cefepime, amoxycillin/clavulanic acid, piperacillin/tazobactam, ertapenem, imipenem, levofloxacin, and trimethoprim/sulfamethoxazole were detected. *Escherichia coli* ATCC25922 was used for quality control.

### DNA Extraction and Sequencing

Genomic DNA was extracted using QIAGEN DNA miniprep kit (QIAGEN, Hilden, Germany). The concentration and purity of the samples were detected by BioDrop μLite+ (BioDrop, Cambridge, UK). The extracted DNA was sent to Hangzhou Digital-Micro Biotechnology Co., Ltd. for sequencing. After library construction, WGS was performed on Illumina Hiseq xTen platform using a 2 × 150-bp paired end (PE) configuration. Sequencing reads were trimmed and *de novo* assembled into contigs using the Shovill pipeline (https://github.com/tseemann/shovill).

### Analysis of WGS Data

MLST, resistance genes, and plasmids were conducted in the Center for Genomic Epidemiology (CGE) by uploading the contigs files obtained from the *de novo* assembly of the WGS data (Larsen et al., 2012). The molecular serotypes of *S. enterica* subsp. *diarizonae* were obtained on the pathogenwatch online (Yoshida et al., 2016). Virulence factors were identified using ABRicate software (V.0.8.10) (https://github.com/tseemann/abricate) by aligned against the Virulence Factors Database (VFDB) (Chen et al., 2016).

## Results

### Source of Isolates and Molecular Epidemiology

In total, three isolates of *S. enterica* subsp. *diarizonae* were isolated during 2015 to 2020; two strains were isolated from 2019 and one from 2020. All of the three strains were derived from fecal specimens. Of these, one isolate was from male and two were from female, and the three patients were all pediatric patients under 1 year old. The serotypes of the three strains were assigned to 61:c:z35, 61:l,v:1,5,7:[z57], and 65:k:z, respectively, while the MLST types were ST1845, ST233, and ST1263, respectively (**Table 1**).

**Table 1:** Source of isolates and molecular epidemiology of *S. enterica* subsp. *diarizonae*.

Isolates	Year of isolation	Specimen	Gender	Age	Serovar	MLST
3198	2019	Fecal	Male	7M10D	61:c:z35	1,845
19CR6061	2019	Fecal	Female	7M17D	61:l,v:1,5,7:[z57]	233
20CR6223	2020	Fecal	Female	3M26D	65:k:z	1,263

### Antimicrobial Susceptibility Test and Resistance Genes

Antibiotic resistance profiles and resistant genes of three isolates of *S. enterica* subsp. *diarizonae* were entirely consistent. All the three isolates were susceptible to ceftriaxone, ceftazidime, cefepime, amoxycillin/clavulanic acid, piperacillin/tazobactam, ertapenem, imipenem, levofloxacin, and trimethoprim/sulfamethoxazole (**Table 2**). No other resistant gene was detected except for *aac(6’)-Iaa*. No gene in any of the resistant plasmids was found in this study.

**Table 2:** MICs for antibiotics of *S. enterica* subsp. *diarizonae* isolates.

Isolates	MICs of antibiotics (µg/ml)								
	Ceftriaxone	Ceftazidime	Cefepime	Amoxicillin/clavulanic acid	Piperacillin/tazobactam	Ertapenem	Imipenem	Levofloxacin	Trimethoprim/sulfamethoxazole
3198	≤0.25	≤0.12	≤0.12	4	≤4	≤0.12	≤0.25	≤0.12	≤1/19
19CR6061	≤0.25	≤0.12	≤0.12	≤2	≤4	≤0.12	≤0.25	≤0.12	≤1/19
20CR6223	≤0.25	≤0.12	≤0.12	≤2	≤4	≤0.12	≤0.25	≤0.12	≤1/19

### Virulence Genes

The distribution of virulence genes of three isolates of *S. enterica* subsp. *diarizonae* was identical. A total of 76 genes were detected, containing 49 genes of Type III Secretion System (T3SS) mediated by SPI-1and SPI-2, 13 genes of adherence (type 1 fimbriae, Agf, and MisL-related genes), 11 genes of iron uptake (Yersiniabactin), two genes of magnesium uptake, and one gene of typhoid toxin (**Table 3** **)**.

**Table 3:** The distribution of virulence genes of *S. enterica* subsp. *diarizonae* isolates.

Classifications	Genes															
T3SS	*avrA*	*invA invB invC invE invF invG invH invI invJ*	*orgA orgB*	*prgH prgJ prgK*	*sicA sicP*	*sipB*	*sigD*	*slrP*	*sopB sopE2*	*sopD2*	*spaO spaP spaQ spaR spaS*	*spiC*	*ssaB ssaC ssaD ssaE ssaG ssaJ ssaL ssaM ssaN ssaR ssaS ssaU ssaV*	*sscA sscB*	*sseB sseE*	*sspA sspB sspC*
Adherence	*csgA csgB csgC csgD csgE csgF csgG*	*fimC fimD fimF fimH fimI*	*misL*													
Magnesium uptake	*mgtB mgtC*															
Iron uptake	*fyuA*	*irp1 irp2*	*ybtA ybtE ybtP ybtQ ybtS ybtT ybtU ybtX*													
Typhoid toxin	*cdtB*															

## Discussion

*Salmonella* causes a variety of symptoms ranging from a mild intestinal infection to life-threatening systemic infections (Chanana et al., 2007), including enteric fever, gastroenteritis, bacteraemia, and systemic infection. All isolates analyzed in this study were derived from fecal specimens, and all the three patients were under 1 year old, which means that *S. enterica* subsp. *diarizonae* mainly causes gastroenteritis, especially in infants. *S. enterica* subsp. *diarizonae* serotypes 43:g,t:- and 8:r:z have been reported in human stools (Guibourdenche et al., 2010), while 48:i:z reported in endocervical tissue and cerebrospinal fluid (Giner-Lamia et al., 2019) and 60:r:z reported in a patient suffering from diarrhea and sepsis (Gerlach et al., 2017). Unlike the studies mentioned above, our current data demonstrated different serotypes (61:c:z35, 61:l,v:1,5,7:[z57] and 65:k:z) of *S. enterica* subsp. *diarizonae* in children’s stools, which were rarely reported in children. However, *S. enterica* subsp. *diarizonae* serotype IIIb_61:I,v:1,5 (Giner-Lamia et al., 2019), strains were reported in wheat grains (Shridhar et al., 2021). MLST is considered to be a well-adopted genotyping method for bacterial epidemiological studies; however, WGS can be used to confirm unusual or unexpected genotyping results, especially in highly recombinogenic pathogens (Aziz et al., 2017). Using WGS, all the three isolates belonged to distinct STs in this study, suggesting that these isolates were not a cluster of epidemic strain. Previous study has reported ST1256 *diarizonae* strains isolated from endocervical tissue and cerebrospinal fluid; other STs of *S. enterica* subsp. *diarizonae* also related to human infections, including ST233, ST430, and ST432 (Giner-Lamia et al., 2019). However, the STs of *S. enterica* subsp. *diarizonae* were ST1845, ST233, and ST1263 in this study. These differences of STs may depend on the geographic location and infection site in which the strains were obtained.

All the three isolates of *S. enterica* subsp. *diarizonae* were susceptible to all the detected antibiotics, and no resistant plasmids were detected in this study. Similarly, two *diarizonae* strains with sequence type ST1256 were found to be susceptible to trimethoprim-sulfametoxazol, quinonoles, aminoglycosides, and most common beta-lactams (Giner-Lamia et al., 2019); a *S. enterica* subsp. *diarizonae* serotype 61:k:1,5 (Giner-Lamia et al., 2019) isolate from urine sample was susceptible to all tested antibiotics (Uelze et al., 2020). However, recent study reported a multidrug-resistant clinical isolate of *S. diarizonae* with a conjugative IncHI2A plasmid (Pan et al., 2021). Therefore, the resistance of *S. enterica* subsp. *diarizonae* needs ongoing attention. In this study, WGS analysis revealed that the resistance gene to aminoglycosides (*aac (6’)-laa*) was present in all isolates; however, the *aac*(*6*′)-*Iaa* gene does not appear to encode for resistance (Leon et al., 2018).

It is known that the critical factor for *Salmonella* survival and establishment of disease in a host is entering into host cells. First, *Salmonella* enters host cells by host invasion pathways, where *Salmonella* adhere to host cells; then, *Salmonella* enters cell invasion pathways, when T3SS secrets a large number of virulence factors (Velge et al., 2012). The most well-studied *Salmonella* pathogenicity islands (SPI) are SPI-1, SPI-2, SPI-3, SPI-4, and SPI-5 (Hensel, 2004). SPI-1 presented in virtually all *Salmonella* isolates, encoded T3SS-1, and related to *Salmonella* invasion of eukaryotic cells; T3SS-2, which was encoded by SPI-2, is required for intracellular proliferation and survival (Coombes et al., 2004). Up to 49 virulence genes of T3SS (mediated by SPI-1and SPI-2) and 13 genes of adherence were identified in the evaluated genomes of the *S. enterica* subsp. *diarizonae* isolates in this study, suggesting that adherence and T3SS played an important role in pathogenicity of *S. enterica* subsp. *diarizonae*. MgtCB can maintain the growth in low Mg2*^+^* and the survival of *Salmonella* in macrophages (Smith et al., 1998). In the present study, *mgtB* and *mgtC*, which are located in SPI-3, were present in all three *S. enterica* subsp. *diarizonae* strains, which indicated that MgtCB contributes to the pathogenicity of *S. enterica* subsp. *diarizonae*. Additionally, *misL* in SPI-3 region and *sopB* in SPI-5 region were present in all *S. enterica* subsp. *diarizonae* strains in this study. Overall, virulence genes of SPI-1, SPI-2, SPI-3, and SPI-5 regions were present in *S. enterica* subsp. *diarizonae* in this study. Distinctly, other studies revealed that *diarizonae* isolates harbor more SPIs, containing SPIs of SPI-1~5, SPI-9, SPI-11, SPI-13, SPI-18, and SPI-21 (Giner-Lamia et al., 2019) and SPIs of SPI-1~5, SPI-9, SPI-12, SPI-13, and SPI-18 (Gerlach et al., 2017). Yersiniabactin is an iron carrier that helps bacteria gain the ability to chelate iron from infected host cells (Perry and Fetherston, 2011). In the present study, the Yersiniabactin genes were present in all isolates, which may pose challenges for clinical treatment. It was known that *cdtB* is strongly associated with persistent and chronic infections of *Salmonella typhi* (Del Bel Belluz et al., 2016). Here, *cdtB* were detected in all three strains of *S. enterica* subsp. *diarizonae* in this study. This finding is in accordance with the study by Gaballa et al. (2021), who revealed the presence of *cdtB* in *S. enterica* subsp. *enterica*, *S. enterica* subsp. *arizonae* and *S. enterica* subsp. *diarizonae* strains. Overall, *S. enterica* subsp. *diarizonae* strains appear to have complex virulence genes, which indicate that much still remains to be learned about the pathogenicity.

## Data Availability Statement

The datasets presented in this study can be found in online repositories. The names of the repository/repositories and accession number(s) can be found below: NCBI; PRJNA740099, SAMN19819870, SAMN19819871, SAMN19819872.

## Ethics Statement

The studies involving human participants were reviewed and approved by The Research and Ethics committee of The Children’s Hospital, Zhejiang University School of Medicine (2021-IRB-031). Written informed consent from the participants’ legal guardian/next of kin was not required to participate in this study in accordance with the national legislation and the institutional requirements.

## Author Contributions

MZ designed the study and drafted the manuscript. QS contributed to the analysis and interpretation of data for the work and revised and critically reviewed the manuscript. XZ performed DNA extraction. LM performed serotyping. YY collected the data. CF carried out antimicrobial susceptibility test. SS designed the study and reviewed the manuscript. All authors contributed to the article and approved the submitted version.

## Conflict of Interest

The authors declare that the research was conducted in the absence of any commercial or financial relationships that could be construed as a potential conflict of interest.

## Publisher’s Note

All claims expressed in this article are solely those of the authors and do not necessarily represent those of their affiliated organizations, or those of the publisher, the editors and the reviewers. Any product that may be evaluated in this article, or claim that may be made by its manufacturer, is not guaranteed or endorsed by the publisher.
